# Analyzing socio-environmental determinants of bone and soft tissue cancer in Indonesia

**DOI:** 10.1186/s12885-024-11974-8

**Published:** 2024-02-14

**Authors:** Yusuf Alam Romadhon, Yuni Prastyo Kurniati, Jumadi Jumadi, Ali Asghar Alesheikh, Aynaz Lotfata

**Affiliations:** 1 Faculty of Medicine, Universitas Muhammadiyah Surakarta, Surakarta, 57162 Indonesia; 2 Centre for Chronical Disease, Universitas Muhammadiyah Surakarta, Surakarta, 57162 Indonesia; 3 Faculty of Geography, Universitas Muhammadiyah Surakarta, Surakarta, 57162 Indonesia; 4 Department of Geospatial Information Systems, Faculty of Geodesy and Geomatics Engineering, K. N. Toosi University of Technology, Tehran, Iran; 5 Department of Pathology, Microbiology, and Immunology, School of Veterinary Medicine, University of California, Davis, USA

**Keywords:** Bone cancer, Soft tissue cancer, Socio-environmental factors, Risk factors

## Abstract

**Background:**

This study is designed to explore the potential impact of individual and environmental residential factors as risk determinants for bone and soft tissue cancers, with a particular focus on the Indonesian context. While it is widely recognized that our living environment can significantly influence cancer development, there has been a notable scarcity of research into how specific living environment characteristics relate to the risk of bone and soft tissue cancers.

**Methods:**

In a cross-sectional study, we analyzed the medical records of oncology patients treated at Prof. Suharso National Referral Orthopedic Hospital. The study aimed to assess tumor malignancy levels and explore the relationships with socio-environmental variables, including gender, distance from the sea, sunrise time, altitude, and population density. Data were gathered in 2020 from diverse sources, including medical records, Google Earth, and local statistical centers. The statistical analyses employed Chi-square and logistic regression techniques with the support of Predictive Analytics SoftWare (PASW) Statistics 18.

**Results:**

Both bivariate and multivariate analyses revealed two significant factors associated with the occurrence of bone and soft tissue cancer. Age exhibited a statistically significant influence (OR of 5.345 and a *p*-value of 0.000 < 0.05), indicating a robust connection between cancer development and age. Additionally, residing within a distance of less than 14 km from the sea significantly affected the likelihood of bone and soft tissue cancers OR 5.604 and *p*-value (0.001 < 0.05).

**Conclusions:**

The study underscores the strong association between age and the development of these cancers, emphasizing the need for heightened vigilance and screening measures in older populations. Moreover, proximity to the sea emerges as another noteworthy factor influencing cancer risk, suggesting potential environmental factors at play. These results highlight the multifaceted nature of cancer causation and underscore the importance of considering socio-environmental variables when assessing cancer risk factors. Such insights can inform more targeted prevention and early detection strategies, ultimately contributing to improved cancer management and patient outcomes.

**Supplementary Information:**

The online version contains supplementary material available at 10.1186/s12885-024-11974-8.

## Introduction

Bone and soft tissue tumors is a term for a group of tumors that affect as the name suggests the bones and soft tissues. When more detailed there are tumor groups from adipocytic tissue, fibroblast and myofibroblast, fibrohistiocytic, vascular, perisitic (perivascular), smooth muscle cells, skeletal muscle, gastrointestinal stroma, chondro-osseous, peripheral nerve sheat, uncertain differentiation, and undifferentiated small cell sarcoma of bone and soft tissue [1, 2]. Osteosarcoma and Ewing sarcoma are the most common bone malignancies in children and adolescents, with an incidence of six new cases per 1000,000 general population per year [3]. Bone and soft tissue sarcomas make up more than 12% of all pediatric malignancies [4]. Soft tissue sarcoma is a rare tumor in adults and accounts for 1% of all malignancies in adults [5]. The distribution of bone and soft tissue tumors may vary by region of residence, but no significant differences are found between musculoskeletal tumors from different regions [6, 7]. There is limited information in the literature on environmental influences on the incidence of bone and soft tissue tumors. There is one piece of literature that states that proximity to industrial centers increases the prevalence of bone tumors in children by 1—3 km. The prevalence is even greater when close to metal production and processing and urban waste-water treatment plants [8].

Indonesia's efforts to achieve significant industrial growth have encountered challenges, but the nation remains resolute in its mission to foster large-scale industrial and economic advancement [9, 10]. The focal point of the industrialization initiative is Java Island, where approximately 80% of the nation's population resides [11]. Industrial development leads to environmental consequences. Increased industrial emissions exacerbate air pollution and improper waste disposal pollutes water sources. These outcomes emphasize the necessity for sustainable solutions, particularly in light of the pollution observed along Java's northern coast [12–16].

Studies have primarily focused on river estuaries and coastal areas to assess pollution levels [17, 18]. Accumulated residential and industrial waste, particularly non-metallic refuse, contributes to pollution in these areas [19]. In addition to activities such as shipping and seafood processing, the growing coastal industries are expected to introduce heavy metal waste into the environment [20, 21]. Oil spills pose an additional threat to the environment, putting harmful substances to organisms [22]. Using rivers as dumping grounds for waste leads to the accumulation of pollution in estuaries and coastal region [23].

Environmental and socio-demographic factors significantly impact the onset and diagnosis of various cancers. In China, the prevalence of cancer types is closely linked to changes in risk factors like diet and pollution [24] In Ethiopia, prevalent cancers frequently encounter delayed diagnoses, especially among females [25]. A study conducted in Australia further underscores the interplay of age and education with cancer diagnosis, highlighting the correlation between low health literacy and advanced-stage cancer detection [26]. These insights underscore the need for improved health literacy and prompt diagnostic interventions.

In a study conducted in the northern Semarang area along the Java coast, the presence of heavy metals in local freshwater sources was associated with a higher likelihood of elevated heavy metal levels in women of reproductive age (OR 3.020, 95% CI = 1.043—8.739) [27]. As found in numerous research, heavy metals contaminate live species in the environment and be consumed by humans in raw or processed foods, such as fish and shellfish [21, 28]. The presence of heavy metals in an environment is linked to the incidence of age-related diseases, including cancer, within the population residing in that environment [29]. Non-metallic pollutants, like polycyclic aromatic hydrocarbons, stimulate enzymes that produce free radicals, thereby elevating the risk of DNA damage, which is one of the pathways to cancer development [30].

Interest in the covariate sun rise—working time, based on an American study, found that the westernmost region in the same time zone had a higher suicide rate than the eastern part. This finding has a similar pattern to the high mortality of people with cancer [31]. There is the term “sun time difference” in the same time zone, which emphasizes that there is a similarity of time related to the political division of time, but there is a difference in sunrise time. In one study, it was found that living in the east compared to the west in the same time zone, based on standard time, both fell into the morning category, but people living in the easternmost region fell into the later sun time category [32]. Whether these covariates have an influence on bone and soft tissue tumor types has not been explored.

The examination of socio-environmental risk factors for bone and soft tissue cancer through the use of statistical models has played a crucial role in unraveling the complexities of this disease. Employing statistical techniques such as Logistic Regression (LR) and Bayesian Regression in biomedical and epidemiological datasets enables the identification of relationships among variables and the interpretation of intricate data sets. Specifically, LR models, like the one utilized by [33] for assessing knowledge about Human Papillomavirus (HPV), are capable of distinguishing between low and high probabilities of an outcome. Meanwhile, studies such as [34] utilized Bayesian Kernel Machine Regression (BKMR) models to assess the impact of intricate mixtures of chemicals on breast cancer risk, taking into account the correlations between chemicals. While the application of LR to analyze cancer risk is not groundbreaking in itself, the novel approach of combining logistic and Bayesian regressions to scrutinize and derive insights from diverse biomedical datasets, particularly when assessing multiple interconnected exposures concurrently, can be considered innovative. The challenge lies in identifying the most suitable approach for a particular dataset and ensuring accurate interpretation of results, as seen in the studies by [35, 36].

While previous research has made significant progress in understanding the biological impact of pollution on non-human ecosystems [37], there is a noticeable research gap concerning its effects on human health, specifically in the context of bone and soft tissue malignancies in Indonesia. Limited studies have explored the correlation between polluted environments and the region's frequency of bone and soft tissue malignancies. To address this gap, the current study investigates the relationship between individual characteristics, geographic location, and the risk of bone and soft tissue malignancies in Indonesia, focusing on patients treated at the Surakarta Orthopaedic Hospital. Tissue samples will be examined at the Pathology Anatomy Laboratory, Faculty of Medicine, Universitas Muhammadiyah Surakarta. The study's objective is to offer insights into the risk factors and root causes of these particular malignancies, thereby enhancing our overall comprehension of how pollution affects human health. This information will also assist healthcare professionals in making informed decisions regarding treatment options and prognostic assessments for patients diagnosed with tumors.

## Materials and methods

### Bone and soft tissue cancer as the outcome variable

**Fig. 1:**
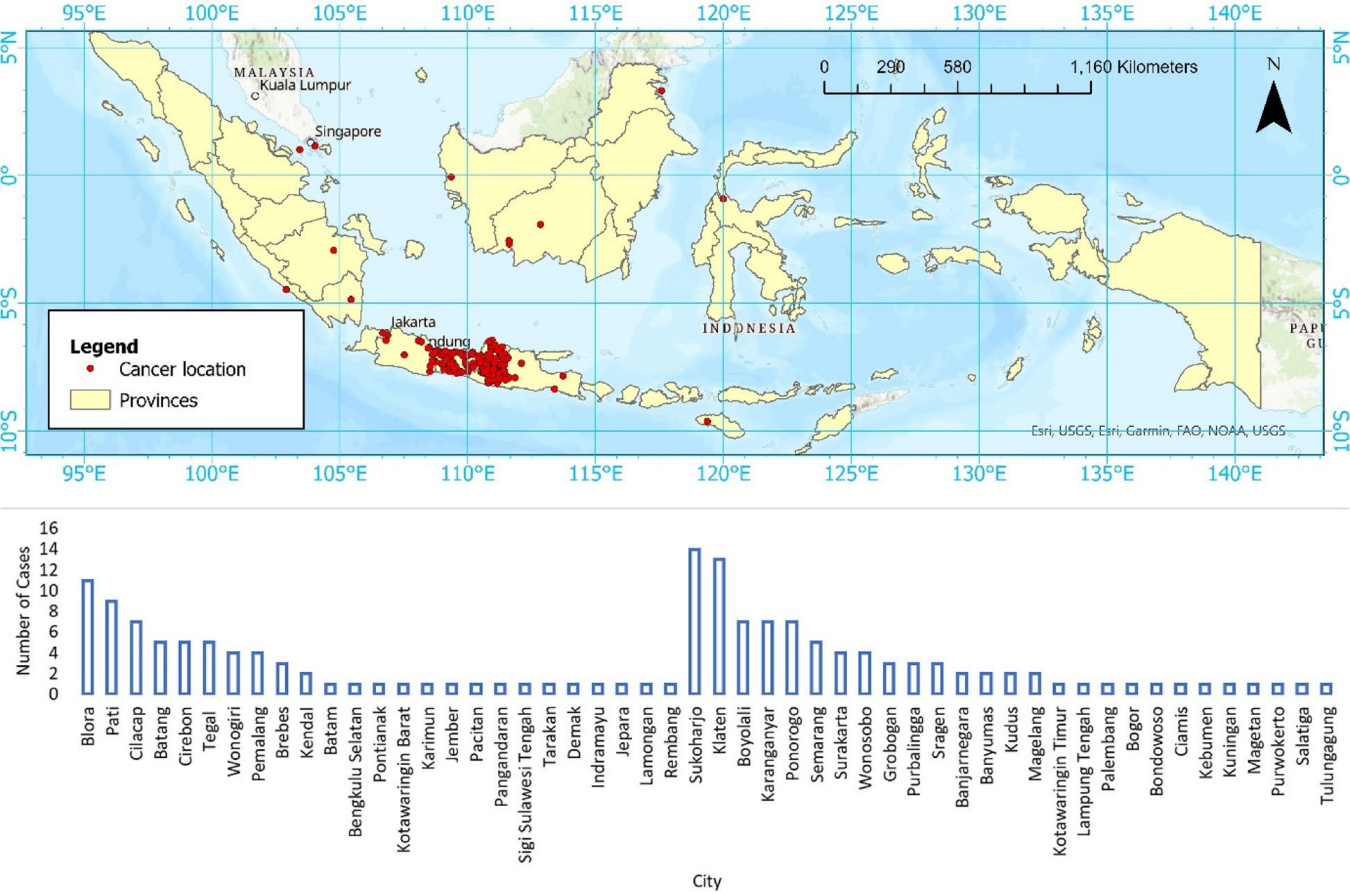
The distribution of cases from 2019 to 2020

A cross-sectional study was carried out by examining medical records from oncology patients at Prof. Suharso National Referral Orthopedic Hospital. The processing and diagnosis of histopathological specimens took place at the Pathology Anatomy Laboratory, Faculty of Medicine, Universitas Muhammadiyah Surakarta. Data spanning from 2019 to 2020, along with their geographic distribution depicted in Fig. 1, were included in the analysis. This study encompasses cases of bone and soft that are categorized into benign or malignant based on WHO criteria, with the soft tissue cancer category.

### Tumor malignancy classification

The histopathology preparations were examined directly by an Anatomical Pathologist (YPK). The data was supplemented with medical records of RSOP Dr. Soeharso Surakarta, adjusted for inclusion and exclusion criteria using the principle of purposive sampling. The preparations taken were from histopathology preparations with a diagnosis of bone and soft tissue tumors according to the WHO classification. The preparations were then sorted out which included primary tumors, not a metastasis. Then, the preparations were also confirmed not to be tumors from other system classifications, or diseases caused by metabolic disorders, infections and or not a 'tumor-like lesion'. Examples of medical records with such histopathological diagnoses are Osteomyelitis, Tuberculosis Infection, Gout, skin cancer, and various cysts. Age and residence data were as written in the medical record. The bone tumors are categorized into "Benign" and "Malignant” with specific diagnosis (Table 1). There are 82 benign cases with various diagnoses like Giant Cell Tumor of Bone and Osteochondroma, and 78 malignant cases, including diagnoses like Osteosarcoma and Plasmacytoma.

**Table 1:** Distribution of diagnoses and categories of benign/malignant bone and soft tissue tumors (*n* = 160). Criteria for diagnosis of benign/malignant division based on WHO criteria for bone and soft tissue tumors

Malignancy Level	Diagnose	Cases	Total
Benign	Giant Cell Tumor of Bone	33	82
	Osteochondroma	15	
	Aneurysmal Bone Cyst	7	
	Fibrous Displasia	7	
	Hemangioma	5	
	Simple Bone Cyst	5	
	Schwannoma	3	
	Neurofibroma	2	
	Angiomyolipoma	1	
	Chondromyxoid Fibroma	1	
	Fibrolipoma	1	
	Osteofibrous Displasia	1	
	Osteoma	1	
Malignant	Osteosarcoma	28	78
	Plasmacytoma	13	
	Malignant Peripheral Nerve Sheath	10	
	Rhabdomyosarcoma	7	
	Lymphoma Maligna	6	
	Chondrosarcoma	4	
	Ewing Sarcoma	3	
	Undifferentiated high-grade pleomorfik Sarcoma	2	
	Soft Tissue Sarcoma	1	
	Adamantinoma	1	
	Fibrosarcoma of Bone	1	
	Glomus Tumor	1	
	Melanoma Maligna	1	

### Covariates

Table 2 provides a summary of the covariates under investigation in the study, organized into three primary categories: Individual Characteristics, Environmental Characteristics, and Demographic Characteristics. The Individual Characteristics includes several variables: age, divided into five distinct age groups; gender, categorized as Female or Male; and Level of Malignancy, classifying tumors as either Non-Malignant or Malignant.

**Table 2:** Illustrating socio-environmental covariates

Variable Type	Variables	Attribute
**Individual Characteristics**	Age	< 10
		11–20
		21–44
		45–59
		60 +
	Sex	Female
		Male
	Type of tumor	Benign
		Malignant
**Environmental Characteristics**	Distance to shoreline	>14 km
		≤14 km
	Sunrise to working time	>2.2 h
		≤ 2.2 h
	Elevation (meter above sea levels)	>30 m
		≤30 m
**Demographical Characteristics**	Population density	>2500/km^2^
		≤2500/km^2^

The Environmental Characteristics encompass various factors, including Distance to Shoreline, categorized as greater than 14 km or less. Another variable is Sunrise to Working Time, categorized based on a threshold of 2.2 h. Finally, Elevation is defined as being 30 m above sea level.

In the category of Demographic Characteristics, the sole considered variable is Population Density, further categorized into two groups based on a threshold of 2,500 individuals per square kilometer.

The study leverages a range of covariates, including age, gender, distance of residence from the sea, local sunrise time relative to working hours (set at 08:00), altitude of the residence above sea level, and population density. Data for these covariates were sourced from multiple avenues to ensure comprehensive and reliable information. Individual characteristics such as age, gender, and place of residence were meticulously gathered from medical records at the Surakarta Orthopedic Hospital. Meanwhile, environmental characteristics like the altitude of the residence and its distance from the sea were extracted from the Google Earth application, a tool that offers geospatial data with high levels of accuracy [38]. Data pertaining to the province's population density was obtained from the local statistical center bureau of the respective province, with the most recent data available from the year 2020 [39]. All the gathered data were in categorical format.

## Data analysis

### Chi-square test

Chi-square test is a statistical method used to test hypotheses expressed with Pearson's Chi-square statistic. It is employed for bivariate analysis, examining the relationship between two dichotomous variables [40]. In this test, the conditions, typically exposure (risk/predictor), and outcome are arranged in a 2 × 2 table format, a commonly used format in this analysis [41, 42] Crude Odds Ratio (OR) is a statistical measure used to assess risk by comparing the proportions (percentage/prevalence) of the exposed group to the non-exposed group. The association is analyzed bivariately, involving one independent variable and one dependent variable. The odds ratio can be calculated using the chi-square test formula (Eq. 1).

1 $$95\% CI=OR \pm 1.96* \sqrt{\frac{1}{a}+ \frac{1}{b}+ \frac{1}{c}+ \frac{1}{d}}$$

Here, *a*, *b*, *c*, and *d* represent the counts in the 2 × 2 table cells.

### Odds ratio

Odds ratio (OR) is a crucial statistical measure extensively used in clinical research and decision-making. It quantifies the association between exposure (risk/predictor) and outcome by organizing data in tabular form. The calculation involves the comparison of the odds of an event occurring in the exposed group to the odds in the non-exposed group (Eq. 2).

2 $$OR= \frac{ad}{bc}$$

where *a*, *b*, *c*, and *d* are the counts in the 2 × 2 table cells representing exposed and non-exposed groups.

### Multivariate logistic regression

Multivariate logistic regression is a statistical analysis model that estimates the relationship between a dependent variable (such as clinical outcomes or diseases) and more than one independent variable (predictors/risks). In medical research, regression analysis applications commonly include linear regression for continuous variables and logistic regression for binary dichotomous variables [43, 44]. Logistic regression is widely used to investigate associations between risk exposure and binary outcomes. For instance, this model can be used to estimate the effect of specific clinical characteristics (multiple factors like obesity, smoking, history of stroke, exercise, or diet) on a particular health condition like cardiovascular events, mortality, or hospital admission [45]. Logistic regression is regularly employed to estimate the effect of specific independent variables, adjusted to control confounding factors in epidemiological studies [46, 47]. The logistic regression probability function is expressed as Eq. 3.

3 $${P\left(Y=1|{X}_{1}\right)= \frac{1}{1+ {e}^{- ({\beta }_{0}+{\beta }_{1}+{X}_{1} )}}}$$

where ***Y*** is the binary outcome variable, ***X***_***1***_ is the predictor variable, ***β***_***0***_ is the intercept, and ***β***_***1***_ is the coefficient for ***X***_***1.***_ The odds ratio from logistic regression is calculated as Eq. 4.

4 $$OR= {e}^{{\beta }_{1}}$$

In logistic regression, the odds ratio is represented as ${e}^{\beta 1}$, indicating how the odds of the event (Y = 1) change with a unit change in the predictor variable ***X***_***1***_. The sample size in logistic regression is 10 times the number of parameters [48, 49] or 20 times [50]. Logistic regression analysis uses the maximum likelihood estimator, although this analysis does not require the assumption of data normality, the assumptions of multicollinearity and outliers need to be checked [48, 49, 51]. Multicollinearity can be seen from the correlation between variables < 0.90 [52], and outliers are seen when the normalized residual value is above ± 3.3 [53].The next stage of analysis in logistic regression is to check the goodness of the overall model with the Log Likelihood Ratio test where the *p*-value < 0.05 [54]. In addition, the Hosmer and Lemeshow test can be used to see if the data fits the model. It is expected that the *p*-value of this test is > 0.05 to accept the hypothesis that the data fits the model [48, 49, 51]. The Pseudo R square test known as Nagelkerke R square explains the variation in tumor data that can be explained by inndependent variables variations. To test the model prediction performance we use the classification matrix (classification accuracy) [48, 49] and ROC Curve [55] while 0.70—0.80 acceptable and above > 0.80 is satisfactory in cancer studies ( citaion please from studies show these numbers as you mentioned here).

The statistical analyses described, including the Chi-square test and multivariate logistic regression, were performed using PASW Statistics 18.

## Results

### Overview of the bone and soft tissue cancer cases

A total of 160 research subjects were involved in this study. Two-thirds of the cases were under 40, with an equal proportion of men and women. Almost half of the tumor cases were diagnosed with malignancy, and three-quarters of people with diagnosed cancer resided at a distance greater than 14 km from the sea. Nearly all cases lived in an area where the local sunrise time differed from working hours [08:00] by more than 2.2 h, and more than three-quarters of the respondents lived in areas with a population density of less than 2,500 people/km^2^ (Table 3).

**Table 3:** Characteristics of Patient (*n* = 160)

Variable Type	Variables	Attribute	Σ	%
**Individual Characteristics**	Age (years)	< 40	106	66,3
		40 + +	54	33,8
	Sex	Female	77	48,1
		Male	83	51,9
	Type of tumors	Benign	82	51,3
		Malignant	78	48,8
**Environmental Characteristics**	Distance to shoreline	>14 km	119	74,4
		≤14 km	41	25,6
	Sunrise to working time	>2.2 h	148	92,5
		≤2.2 h	12	7,5
	Elevation (masl)	>30 masl	121	75,6
		≤30 masl	39	24,4
**Demographical Characteristics**	Population density	>2500/km^2^	124	77,5
		≤2500/km^2^	36	22,5

**Fig. 2:**
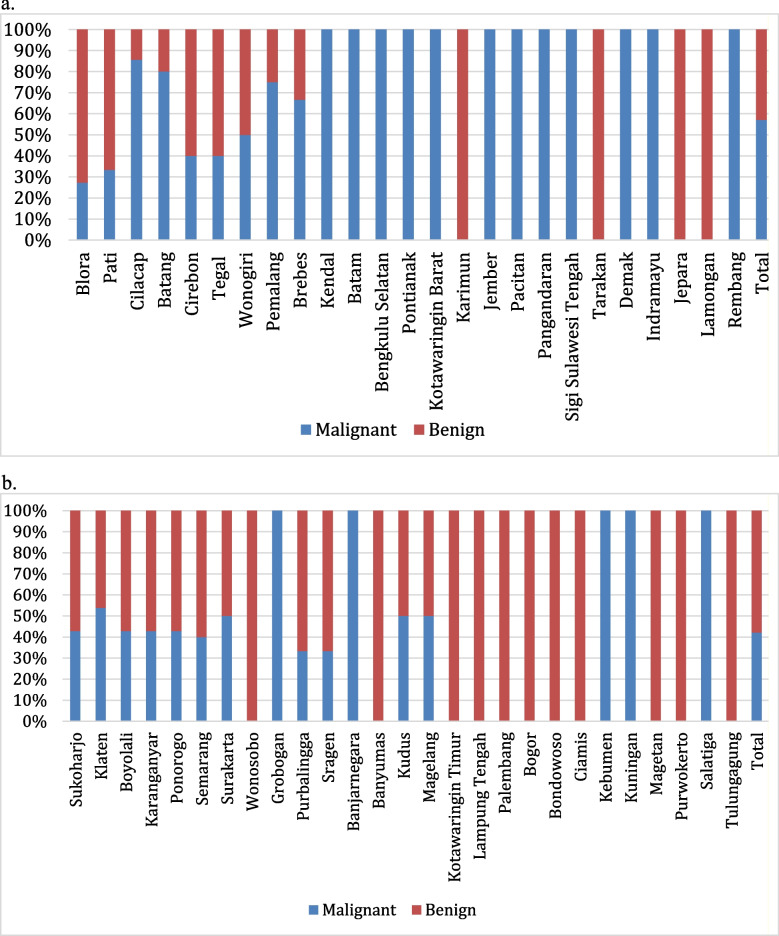
Distribution of Cases Origin. a. Chart illustrating the comparison of the proportion of malignant vs. benign types of bone and soft tissue tumors in areas close to the coast. b. Chart illustrating the comparison of the proportion of malignant vs. benign types of bone and soft tissue tumors in the relatively central region of the island

Table 4 presents data on the occurrence of benign and malignant cases in relation to their proximity to coastal areas. It is categorized into two groups: cases relatively close to the coast and cases in the middle of an island. For the areas relatively close to the coast, there are 30 benign cases (43%) and 40 malignant cases (57%), totaling 70 cases. In contrast, in the middle of the island, there are 52 benign cases (58%) and 38 malignant cases (42%), with a total of 90 cases. This suggests a higher proportion of benign cases in more inland areas compared to coastal areas. Figure 2 provides the distribution of cases and their corresponding cases of malignant and benign, totaling each city's cases and categorizing them based on their proximity to the coastline. This figure shows a majority of the cases listed are close to the coastline, such as Blora, Pati, and Cilacap, with Blora having highest malignancy level.

**Table 4:** Illustrating proportion of benign vs malignant bone and soft tissue tumors between nearshore vs mid-island relative regions

Proximity to coastal area	benign		malignant		Total
	Cases	%	Cases	%	
Coastal	30	43	40	57	70
Inland	52	58	38	42	90

### Socio-environmental factors of bone and soft tissue cancer incident

According to the bivariate analysis, two risk factors significantly influenced bone and soft tissue malignancy levels out of the five independent variables examined. These risk factors included being over 40, which had a 3,919 times greater risk of malignancy compared to lower age groups [OR = 3,919, *p* = 0.000]. The other significant factor was the residential distance from the sea, with those living within 14 km having a 5,749 times higher risk of malignancy than those living farther away [OR = 5,749, *p* = 0.000]. Living at an altitude of < / = 30 masl, in bivariate analysis had a risk of 2.654 times compared to living in a higher area [OR = 2.654, *p* = 0.011], but in logistic regression analysis did not show statistical significance [OR = 1.417, *p* = 0.503] (Table 5).

**Table 5:** Goodness of fit

.-2 Loglikelihood	*P*-value	Uji Hosmer and Lemeshow			R Square nagelkerke
		Chi Square	df	*p*-value	
183,1	0.000	12,614	7	0,082	0.286

Checking the assumptions of logistic regression, Pallant [51] shows satisfactory results where there is no correlation between variables above 0.90. In addition, the standardized residual value is below 3.3, indicating no outlier data. Furthermore, the overall model is accepted with Log likelihood where the *p*-value of -2 Log Likelihood test is 0.000 < 0.05 which indicates that by including independent variables in the model, there is a significant variable on the dependent variable (tumor). Furthermore, the Hosmer and Lemeshow test shows satisfactory results where the Null Hypothesis (H0) of this test is that the data fits the model. This test is Chi square distributed with df = 7 and the *p*-value of the Hosmer and Lemeshow test is 0.082 > 0.05, so accept Ho which means that empirical data can explain the model. Pallant [51] explained that Nagelkerke's R square with variation in number (malignant or benign) was 28.6%.

Based on the results of multivariate testing with logistic regression, it can be seen that there are 2 (two) significant variables, namely age with an OR of 5.345 and a *p*-value of 0.000 < 0.05. This indicates that the risk of patients with suai above 40 years of age to develop malignant tumors will increase 5.345 times compared to patients under the age of 40 years. The second variable is distance to shoreline with OR 5.604 and *p*-value (0.001 < 0.05). The risk of patients with distance to shoreline above 14 km will increase 5.604 times. While other variables namely gender, local sunrise to working hour, elevation and population density are not significant (see Table 6).

**Table 6:** Bivariate and multivariate analyses contributed to environmental characteristics as a risk factor for bone and soft tissue malignancy

Variable	Malignancy rate of bone and soft tissue tumors		Bivariate Analysis		Multivariate Analysis	
	Benign (*n* =)/Σ[%]	Malignant (*n* =)/ Σ[%]	OR	*P*	OR	*P*
**Age**						
<years (ref)	66 (62.3%)	40 (37.7%)	3.919	0.000	5.345	0.000
≤40 years	16 (29.6%)	38 (70.4%)				
**Gender**						
Female (ref)	40 (51.9%)	37 (48.1%)	1.055	0.865	1.013	0.971
Male	42 (50.6%)	41 (49.4%)				
**Distance to shoreline**						
>14 km (ref)	10 (24.4%)	31 (75.6%)	5.749	0.000	5.604	0.001
≤14 km	72 (60.5%)	47 (39.5%)				
**Local sunrise to working hour**						
>2.2h (ref)	6 (50%)	6 (50%)	1.056	0.928	0.636	0.548
≤2.2 h	76 (51.4%)	72 (48.6%)				
**Elevation**						
>masl (ref)	69 (57%)	52 (43%)	2.654	0.011	1.417	0.503
≤30 masl	13 (33.3%)	26 (66.7%)				
**Population density**						
>km^2^	61 (49.2%)	63 (50.8%)	0.692	0.335	0.543	0.175
≤2500/km^2^ (ref)	21 (58.3%)	15 (41.7%)				

aOR age and distance to shoreline adjusted for local sunrise to working hour, elevation and population density

The final part of the logistic regression model is to test how well the resulting regression model predicts between the observed data and its prediction. Of the 82 patients with non-malignant tumor status (coding 0), there were 56 patients or 68.3% correctly classified as non-malignant tumor patients. And of the 78 patients with malignant tumor status (coding 1), there were 57 patients or 73.1% correctly classified as malignant tumor patients. Overall, the percentage of prediction accuracy is 70.6%. Furthermore, the ROC Curve analysis showed 0.707 which according to Hosmer and Lemeshow [55] is between 0.70—0.80 acceptable (Table 7).

**Table 7:** Classification Accuracy dan ROC Analysis

Observed		Predicted			ROC Area
		Tumor type		Percentage Correct	
		benign	malignant		
Type of tumor	0	56	26	68.3	0.707
	1	21	57	73.1	
Overall Percentage				70.6	

## Discussion

In summary, the major finding of this study underscores that individuals aged 40 /older and who live close to the coastline face an increased risk of developing bone and soft tissue cancer. In this study, elderly people had a higher risk of experiencing bone and soft tissue malignancies. Malignant bone tumors, such as osteosarcoma, have a bimodal age distribution, with peaks in pediatric and older adult populations [56]. The global incidence of primary malignant bone tumors shows two peaks at 10–20 years and a steady increase from 40 to 80 years, with males being affected more frequently than females [57]. Aging is associated with an increase in cancer cases and fatalities. The chances of cancer increase exponentially starting from middle age, but there is a decline in reported cases above the age of 80 [58, 59].

**Fig. 3:**
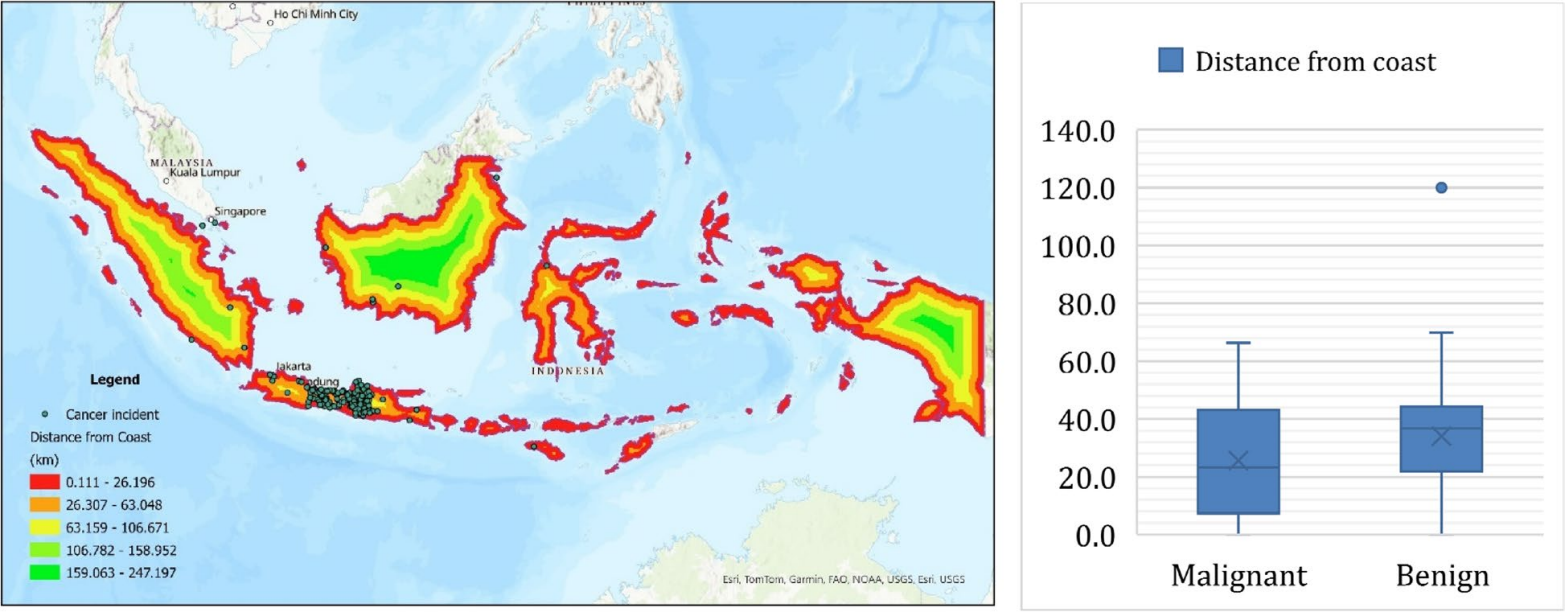
Relation of cases and distance to coastline

Figure 3 illustrates relationship between the malignancy level of cancer and the distance from the coastline. It compares the average, maximum, and minimum distances from the coast for both benign and malignant cancer cases. For benign cases, the average distance from the coastline is 34.04 km, with a maximum distance of approximately 120 km and a minimum of 0 km. In contrast, malignant cases have a closer average distance to the coast at 25.59 km, with a maximum distance of around 66.33 km and a minimum of 0 km. This data suggests that malignant cancer cases tend to be located closer to the coastline compared to benign cases.

These types of tumors exhibit a wide range of histological characteristics [60]. Aging increases the prevalence of malignancy in general and the risk of metastasis [61, 62]. Even for bone tumors typically associated with adolescents and young adults, the incidence escalates with age, along with the risk of malignancy [63]. Notably, individuals over the age of 35 have a 4.13 times higher risk [OR = 4.13 (95% CI 3.64–4.68) p0.01] of developing Rhabdomyosarcoma, as indicated by a national study involving 4,787 patients in the United States. This underscores the significant impact of age on cancer risk [64].

Environmental factors play a crucial role in the development of cancer, particularly in bone and soft tissue [65]. Exposure to carcinogenic chemicals varies based on geographical factors, and within each region, unique genetic traits and demographic variations may predispose individuals to different cancer types. Ethnic dominance within regions can further influence these disparities [66].

**Fig. 4:**
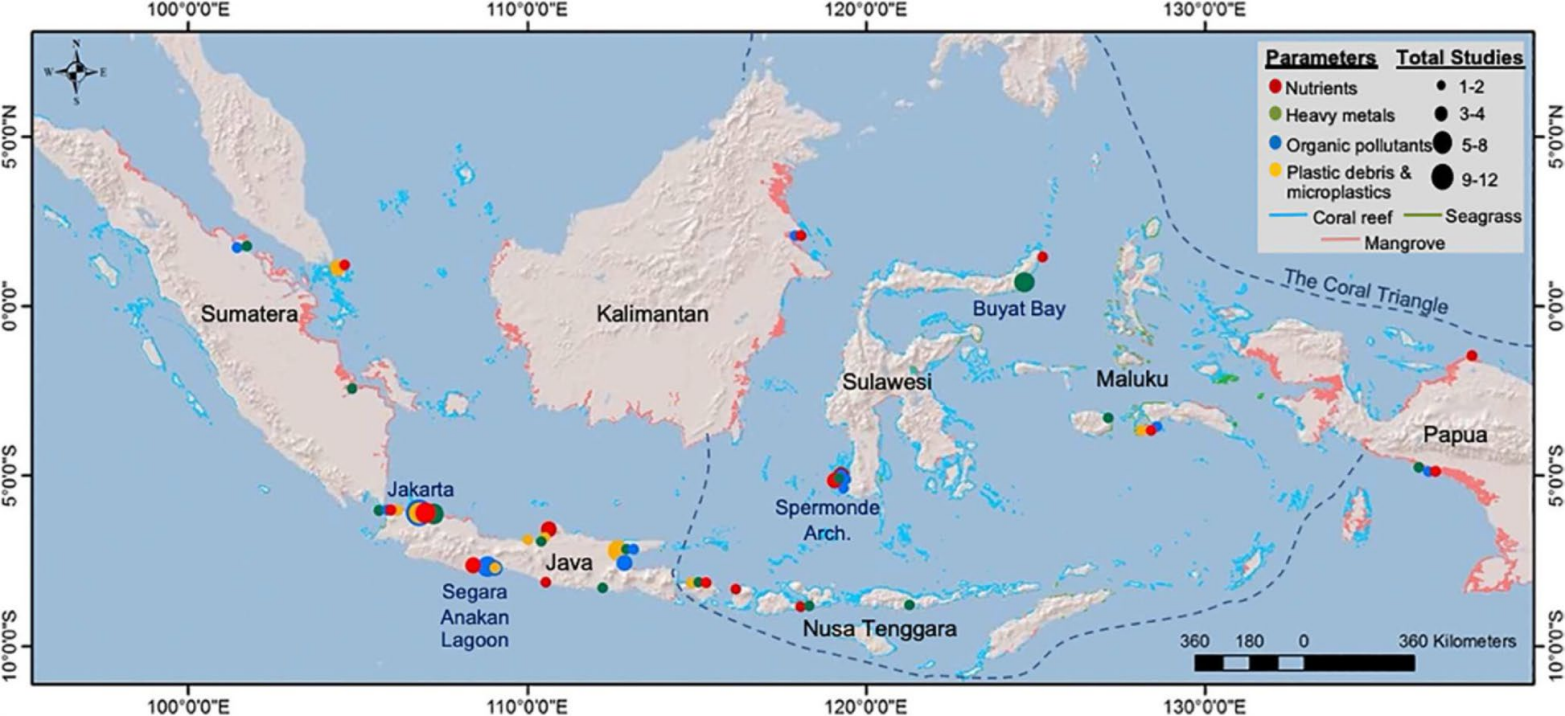
Intersections with areas that have relatively high levels of environmental pollution in Indonesia [67]

The stereotype of the area where people with malignant bone and soft tissue tumors live in this study, from satellite images, shows patterns of agricultural areas, dense residential areas and river estuaries (Fig. 4). The use of organophosphate pesticides is common in agricultural areas. In a study in West Java by taking several samples along river flows, it was found that the concentration of organophosphates in the water tended to be relatively high in areas close to rice fields, as did the levels in river sediment. Although these organophosphate compounds are degraded over time varying from 6 h to weeks [68]. Organophosphate compounds such as malathion and diazinon increase the risk of breast, ovarian and thyroid cancer, but not non-Hodgkin lymphoma (a type of soft tissue tumor) [69]. However, IARC includes diazinon as a risk factor for lymphoma cancer with sufficient or limited evidence in humans [70]. Research evaluating the effect of exposure to organochloride compounds on genotoxic activity in school children in Mexico. Technically, this research evaluates the level of exposure to organochloride compounds by examining the levels of compounds in hair roots, while evaluating gene damage by examining buccal cells. Research findings prove that exposure to organochloride compounds increases the risk of genotoxicity [71]. Large-scale research with a 2-year follow-up interval in agricultural areas found that among the pesticide compounds that had a significant trend in increasing the risk of lymphoma included DDT, lindane and fumigants [72].

A meta-analysis study of past data in England showed an increase in the prevalence of skeletal malignancies from 0.06% in the medieval era to 0.36 in the industrialization era (*p* < 0.001). Age showed a strong relationship with malignancy (*p* = 0.003) but not with sex (*p* = 0.464) [73]. The similarities with the current study are the rise of industrialization and its influence on skeletal malignancies, and age has a strong correlation with malignancy.

The study also highlights the role of exposure to carcinogens, such as heavy metals, in inducing oxidative stress and increasing the risk of DNA, lipid, and protein damage [74]. Notably, a study in Iraq observed significantly higher levels of heavy metals in the blood plasma of cancer patients compared to the non-cancer population, further highlighting the link between environmental factors and cancer risk [75].

Chronic low-grade systemic inflammation in the presence of heavy metal exposure is an additional mechanism that heightens the risk of cancer. A comparable study in Spain used proximity to pollution sources as a criterion for estimating risk. The Spanish study defined proximity as a distance of 2 km or less from specified industrial zones or cities, and it found an elevated risk of leukemia, neuroblastoma, kidney tumors, and bone tumors in these areas [76].

The findings of our study open the door to new research directions. A study in China, for instance, established a connection between high soil pollution with heavy metals, particularly Cadmium, and the risk of stomach cancer [77]. These findings suggest that environmental factors, in particular, proximity to pollution sources and exposure to heavy metals, are significant contributors to the risk of various types of cancer and underscore the importance of further research in this area.

### Limitations and future studies

Regarding the limitations, it is important to highlight the cross-sectional design employed in this investigation, which inherently imposes constraints on our capacity to demonstrate a causal relationship between the identified risk factors and the occurrence of bone and soft tissue cancer. Although notable correlations were identified, a longitudinal approach would enhance our comprehension of the temporal order and causal connections. Finally, despite the inclusion of several environmental and demographic covariates, it is important to acknowledge the possibility of unmeasured confounding variables, such as lifestyle habits (e.g., smoking, food, and exercise), that were not taken into consideration and may have a possible influence on the findings of the study.

To enhance the scope of research, it is recommended to undertake a longitudinal investigation that monitors individuals over an extended period. This approach would provide a more robust determination of causation pertaining to the discovered risk variables and their impact on cancer outcomes. Expanding the sample size to include a broader range of participants from several hospitals or locations would contribute to a more comprehensive perspective and improve the applicability of the results. Furthermore, the inclusion of additional covariates, such as lifestyle characteristics and potential environmental exposures, has the potential to enhance our comprehension of the intricate interactions among various factors that influence the susceptibility to bone and soft tissue cancer. These advancements possess the potential to not only enhance the scientific validity of the research but also have practical consequences for focused preventative initiatives.

## Conclusion

Individuals aged 40 and older residing within a 14-km proximity to coastal regions are identified as significant risk factors for bone and soft tissue cancers. These findings have significant implications for healthcare professionals and public health experts, underscoring the pivotal role of demographic and environmental determinants in the comprehensive evaluation of cancer risk and the development of tailored preventive strategies. Nonetheless, further comprehensive research is essential to delve more profoundly into these findings, exploring supplementary contributory factors and potential interactive dynamics that underpin the development of bone and soft tissue cancers. A profound understanding of the fundamental causes of these malignancies can significantly enhance the processes of early detection, risk assessment, and the execution of precisely targeted intervention strategies, thereby ameliorating patient outcomes and alleviating the societal burden associated with these diseases.

### Supplementary Information

**Additional file 1: Table A. **Variable Correlation Matrix.

## Data Availability

The original data supporting these findings are available at any time upon request to the corresponding author.
