# Supplementary material for: Analyzing socio-environmental determinants of bone and soft tissue cancer in Indonesia
Source: BMC Cancer. 2024 Feb 14;24:206. doi: 10.1186/s12885-024-11974-8 (PMC10865616; doi:10.1186/s12885-024-11974-8)
Supplement: Supplementary file 1 — Additional file 1: Table A. Variable Correlation Matrix. [file 12885_2024_11974_MOESM1_ESM.docx]

Appendix

**Table A.** Variable Correlation Matrix

|  | Constant | Age(1) | Gender(1) | Sunrise Interval (1) | Elevation(1) | Population Density(1) | Distance to Coast(1) |
| --- | --- | --- | --- | --- | --- | --- | --- |
| Constant | 1.000 | -.449 | -.544 | -.066 | -.107 | -.191 | -.256 |
| Age (1) | -.449 | 1.000 | -.011 | -.011 | .051 | -.114 | .177 |
| Gender(1) | -.544 | -.011 | 1.000 | .046 | -.114 | -.029 | .029 |
| Sunrise Interval (1) | -.066 | -.011 | .046 | 1.000 | -.224 | -.052 | -.048 |
| Elevation (1) | -.107 | .051 | -.114 | -.224 | 1.000 | -.003 | -.465 |
| Population Density (1) | -.191 | -.114 | -.029 | -.052 | -.003 | 1.000 | -.057 |
| Distance to Coast (1) | -.256 | .177 | .029 | -.048 | -.465 | -.057 | 1.000 |
